# Autism-Related *Cc2d1a* Heterozygous Mice: Increased Levels of miRNAs Retained in DNA/RNA Hybrid Profiles (R-Loop)

**DOI:** 10.3390/biom14091183

**Published:** 2024-09-20

**Authors:** Elif Funda Sener, Halime Dana, Reyhan Tahtasakal, Serpil Taheri, Minoo Rassoulzadegan

**Affiliations:** 1 Genome and Stem Cell Center (GENKOK), Erciyes University, 38039 Kayseri, Türkiye; genkokhalime@gmail.com (H.D.); rreyhan44@gmail.com (R.T.); serpiltaheri@hotmail.com (S.T.); 2 Department of Medical Biology, Medical Faculty, Erciyes University, 38039 Kayseri, Türkiye; 3 The National Institute of Health and Medical Research (INSERM)-Centre National de la Recherche Scientifique (CNRS), Université Côte d’Azur, Inserm, 06000 Nice, France

**Keywords:** *Cc2d1a*, autism, microRNA, transcripts, DNA/RNA hybrid, non-Mendelian heredity

## Abstract

Autism spectrum disorder (ASD) is a complex neurodevelopmental disorder with a highly variable expression of phenotypes (restricted interest or activity and repetitive behavior in communication and social interactions), genes (mutation), markers (alteration of transcription) and pathways. Loss of function of the *CC2D1A* gene appears to primarily affect the brain, leading to a range of behavioral problems in humans. In our study published in 2020, we found that the expressions of miR-19a-3p, miR-361-5p, miR-150-5p, miR-3613-3p, miR-126-3p and miR-499a-5p were downregulated in the serum samples of autistic patients, their families and mouse models (*Cc2d1a* +/− and valproic acid treated males). Here, acquired non-Mendelian hereditary character in a genetically defined mouse model of autism (*Cc2d1a* +/−) correlates with the transcriptional alteration of five miRNAs. We seek to test the hypothesis that miRNA levels vary by changes in RNA/DNA structure during development, thereby creating transcription alteration and cell memory. Behavioral tests were conducted on the offspring of *Cc2d1a* (+/−) mutant and control mice, such as novel object, social interaction, marble burying and tail suspension behavior. Two RNA fractions were isolated from mouse hippocampal tissues and sperm cells via standard TRIzol extraction: free RNA and the fraction of RNA bound to DNA in the form of a DNA/RNA hybrid (R-loop). The expression levels of miR-19a-3p, miR-361-5p, miR-150-5p, miR-126-3p and miR-499a-5p were investigated by quantitative real-time RT-PCR. We report differences in the distribution of five miRNAs in the hippocampus between male and female mice, particularly in colonies of *Cc2d1a* (+/−) mice. Furthermore, the number of miRNAs engaged in the DNA/RNA hybrid fraction is generally higher in the mutant pedigree than in the control group. On the other hand, in sperm, both fractions are at lower levels than in controls. R-loops contribute to the physiology and pathology of organisms including human disease. Here, we report a variation in five miRNA levels between gender and tissue. Our results suggest that the transcription levels of these five miRNAs are directly regulated by their RNA.

## 1. Introduction

Autism spectrum disorder (ASD) is one of the most common neurodevelopmental disorders, affecting approximately 1 in 36 children [1]. ASD affects four times more boys than girls and appears early, with an average age of diagnosis before 3 years [2]. Diagnosis of ASD often reveals various problems such as gastrointestinal system abnormalities, epilepsy, intellectual disability (ID), sleep and motor difficulties [3]. These disorders result from complex interactions between environmental and genetics factors, with a heritability ranging from 40 to 80% [4]. ASD risk factors strongly influence synapse connectivity [5,6].

A large homozygous deletion has been reported in the *CC2D1A* gene, and loss of the gene is associated with non-syndromic disease (ID) in human patients, suggesting that *Cc2d1a* fulfills its functions in the brain. *CC2D1A* protein is a transcription factor and suppressor of the *HTR1A* gene. In our previous study, the expression levels of the *CC2D1A* transcripts (q-PCR) were increased in ASD patients without mutation in autism-linked known genes compared to controls [3]. Mice lacking the *Cc2d1a* gene develop normally during the uterine period but die immediately after birth due to swallowing and respiratory deficiencies. Tissues-specific (hypothalamus) *Cc2d1a* in conditional knock-out mice exhibit learning, memory and social deficits, hyperactivity, repetitive behaviors and anxiety, all of which are key features of autism and ID [7,8]. These results confirm that *Cc2d1a* is one of the new candidate genes for ASD. The *Cc2d1a* heterozygous mouse model showed sex-specific differences and non-Mendelian heritable changes transferred to offspring, even those with a wild-type genotype [6,7,8,9].

Several studies revealed various RNA populations in sperm [10,11,12], but only recently was the possible presence of DNA–RNA hybrids (R-loops) reported. It has been shown that a significant portion of RNA is actually made up of complexes similar to the R-loop, in the form of three-chain structures consisting of an RNA–DNA hybrid and a single strand of DNA [13,14]. Since the discovery of R-loops, one of the most abundant non-B-DNA structures in mammalian genomes [15], various types of biological functions and consequences have been characterized, including their regulatory potential [16]. During spermatogenesis, the histone–protamine transition ensures the integrity of paternal DNA in the sperm head. An incomplete transition from histone to protamine leaves residual histones-associated genomic regions, leading to an open chromatin state and DNA–RNA interactions [17]. Similarly, R-loops bring together canonical histone modifications, capable of influencing DNA–protein interactions, and reconstructing a specific spatial landscape [15]. Additionally, the three-stranded nature of R-loops affects the broader chromatin conformation of the sperm genome and may therefore play a locally antagonistic role to protamine tight packaging.

MicroRNAs (miRNAs) are small non-coding RNA molecules consisting of about 22 nucleotides that act as post-transcriptional regulators of gene expression [18,19]. In mammalian cells, miRNAs were discovered to create double-strand RNA by base pairing with complementary sequences within messenger RNA (mRNA) molecules, which often results in inactivation of transcripts through translational suppression [20,21]. In a previous study we conducted, the expression levels of six miRNAs (miR-19a-3p, miR-361-5p, miR-3613-3p, miR-15a-5p, miR-126-3p and miR-499a-5p) were downregulated in blood serum levels of patients with autism and in various tissues (brain, sperm and blood) in mouse models [7]. Based on this study, we attempt to investigate the heritable downregulation of these six miRNAs in autism with the *Cc2d1a* heterozygous mouse model. In this study, our objective was to discover the molecular differences that participate in the downregulations of the expression profile of five of six miRNAs (miR-19a-3p, miR-361-5p, miR-150a-5p, miR-126-3p and miR-499a-5p) identified in the cohort of autistic patients and their families compared to controls (miR-3613-3p does not exist in mouse genome). Here, we fractionated cellular RNAs using two different isolation methods: extraction of free RNA with the standard TRIzol method and isolation of the DNA-associated RNA fraction as a DNA/RNA hybrid (R-loop) from blood, hippocampal and sperm tissues of *Cc2d1a* (+/−) and control mice. By comparing the levels of these miRNAs, we found differences in the fraction of RNA attached to DNA between sexes and tissues, suggesting that these changes would affect the expression levels. We suggest that miRNAs involved in the R-loop harbor cell memory in the development.

## 2. Material and Methods

### 2.1. Preparing the Animal Groups

*Cc2d1a* (+/−) mice were purchased from the Jackson Laboratory and crossed with the *Balb-C* background for more than twenty generations. All animals were maintained on a 12-h light/12-h dark cycle (lights on from 06:00 to 18:00), under standard housing conditions (21 ± 1 °C, 40–50% humidity, food and water ad libitum) so that their circadian rhythms were not disrupted. This process managed by the Transgenic Unit Facility Staff of GENKOK. All efforts were made to minimize animal suffering and to reduce the number of animals used in the study. Before starting the experiments, the mice were allowed to acclimatize to the new environment for at least one week. Animals were housed in groups of 4–5 mice per cage for time period of 8 weeks. The control group consisted of wild-type (WT) *Balb-C* mice. Two groups of mice were generated in the study. Mice were bred using *Balb-C* WT females and *Cc2d1a* (+/−) males in group 1 (G1). Mice were bred using *Cc2d1a* (+/−) females and *Cc2d1a* (+/−) males in group 2 (G2). *Cc2d1a* (+/−) and (+/+) genotypes were identified using PCR according to the instructions with the three different oligonucleotides. Heterozygotes were selected via PCR genotyping according to the instructions for the corresponding Jackson Laboratory primers (*Ccd1a*-M1: 5′-GTG CGA GGC CAG AGG CCA CTT CTG-3′, *Ccd1a*-M2: 5′-GAC CCT GAG AGA GCT CCT GAG AGC-3′, *Ccd1a*-M3: 5′-TT CCC ACC TCT TCT GGC CCA GAG G-3′). The PCR products were evaluated with agarose gel electrophoresis [9]. Ten male and ten female (+/−) genotyped mice were selected from the nail tissues of each animal in the resulting F1 generation. Parents and (+/−) and (+/+) genotyped two-month-old males and female littermates were used for behavioral studies of tail suspension, marble and novel object recognition test. Ten male and ten female animals at the same age were used in each behavior test. Before the behavioral experiments, mice were transported to the experimental room and left for 5–10 min for habituation. This study was approved by the Animal Ethics Committee of Erciyes University (9 September 2020, 20/124).

### 2.2. Behavior Tests

All tests were performed under normal lighting in the light phase. Care was taken to minimize pain or discomfort for the animals. We started with 10 males and females in each cohort. Each mouse underwent a single test daily between 10:00 and 16:00 h. To minimize possible changes, all behavioral tests were performed by the same investigator from the Transgenic unit. Manual scoring of behavior was performed by a trained experimenter blinded to the genotype of the mice. All animal behavior was filmed, tracked and analyzed with “EthoVision 9” software (Noldus, Wageningen, The Netherlands).

The tail suspension test (TST) shows behavioral despair in a stressful situation. The TST was performed in a quiet laboratory. The animal’s immobility time was recorded to determine the depression status [22]. The animal was suspended above the ground by its tail with a tape. By watching recordings with a video camera, the mobility and immobility times of mice were calculated for six minutes. Measurement began after the first two minutes. Total immobility time and immobility latency for each one-minute block were scored. The time from the start of the test until the mice ceased struggling was recorded as latency. The immobility time was used for statistical analysis [9].

The social interaction test was conducted to measure sociability by evaluating general sociability and interest in social novelty. A rectangular three-compartment box was used as described in our previous study [7]. The test mouse was initially placed into a middle compartment for 5 min while the other compartment was left empty. Floor surfaces were wiped with 70% ethanol between tests. Under the parameters found to be connected to the mouse in the chamber, data were generated using the EthoVision system, and statistical analysis was performed along with comparisons to the control group. The accumulated time spent in each compartment and number of visits to the interaction zone were measured to quantify the mice’s social behavior.

The Novel Object Recognition (NOR) task is a well-established test in various animal models and has been performed to assess cognitive ability. The NOR task consisted of habituation, training and probe testing during 5 days of the experiment. In the first trial, we used (first-day acquisition) animals exposed to two similar objects (small orange boxes) in a chamber for 5 min. During the second trial (second-day retention), mice were again exposed to two different objects for 5 min, including a familiar object from the first trial and a novel object (blue box). During each test, the boxes and objects were cleaned with 70% ethanol. The time spent exploring the novel object and the time spent exploring the familiar object were analyzed by researchers blinded to the experiment.

The marble burying test is used to describe anxiety. The experiment was carried out under dim lighting in a quiet room to reduce the influence of anxiety on behavior. Standard glass marbles were washed with mild laboratory detergent, rinsed with distilled, deionized water and dried, then spaced out evenly in five rows of four marbles on top of the bedding. The mice were left in the cage with the marbles for 30 min. Test recording began immediately after the animal was placed in the cage, as far away from the marbles as possible. Numbers of buried marbles were counted and scored as buried if two-thirds of their surface was covered with bedding [22].

### 2.3. Tissue Collection

After the behavioral experiment, mice with *Cc2d1a* (+/+) and *Cc2d1a* (+/−) genotypes were euthanized via cervical dislocation. Sperm and hippocampal tissues were collected. RNA samples with two different methodologies were obtained from these tissues (total RNA isolation and hybrid DNA/RNA isolation). Total RNA was extracted from sperm and hippocampi of *Cc2d1a* heterozygous mice with the TRIzol method and the DNA/RNA hybrid fraction was isolated as hybrid DNA/RNA (R-loop) with a manual kit [14].

### 2.4. Total RNA and Hybrid DNA/RNA Isolation from Hippocampal Tissue

The hippocampus is divided in half and transferred to TriPure (Roche Catalog No: 11667157001, Mannheim, Germany) by adding 200 µL of chloroform. The aqueous phase is transferred to a new tube. The isopropanol is added proportionally to the aqueous phase. After centrifugation, the supernatant is discarded. 75% ethanol is added to the pellet. After centrifugation, the supernatant is discarded, and 50 µL of nuclease-free water are added for dilution. The Zymo Research kit (Catalog No: D7003, USA) was used for hybrid DNA/RNA isolation. Isolation from hippocampal tissue was preformed according to the manufacturer’s protocol. Alternatively, for high concentrations of DNA and RNA, an elution of ≥50 µL was used. Tissues can be mechanically homogenized for optimal extraction efficiency. The samples were stored at −80 °C after isolation.

### 2.5. RNA and Hybrid DNA/RNA Isolation from Sperm Tissue

Sperm was collected from the vas deferens and epididymis of mice. Half of the treated spermatozoa are introduced into a falcon tube for total RNA isolation. The other half of the treated sperm is introduced into another falcon tube for hybrid DNA/RNA isolation. After centrifugation, the supernatant is transferred to a new falcon. The DTT and TRIzol mixture was added and kept on ice for 5 min. Then, 200 µL of chloroform was added. Isopropanol, equal to its own volume, was added to the aqueous phase and 1 ml of 70% ethanol was added. The supernatant is discarded and 50 µL of nuclease-free water is used for dilution. The Zymo Research kit (Catalog No: D7003, USA) was used for hybrid DNA/RNA isolation. Isolation was performed from sperm according to the manufacturer’s protocol. The samples were stored at −80 °C after isolation.

### 2.6. Complementary DNA (cDNA) Synthesis and miRNA Profiling

The amount of cDNA was determined with the Takara cDNA synthesis kit. The reaction mixture was prepared to make cDNA, with 3.75 µL of RNA, 5 µL of mRQ buffer and 1.25 µL of mRQ enzyme kit to make a total volume of 10 µL. The reverse transcription step was performed at 37 °C for 60 min, followed by 5 min at 85 °C. The cDNA was stored at 4 °C until use. The expression levels of 5 miRNA (miR-19a-3p, miR-361-5p, miR-150-5p, miR-126 and miR-499a-5p) were measured using a chain reaction by real-time quantitative polymerase (qRT-PCR). The TAKARA SYBR^®^ Green PCR Kit (Catalog No.: 638316, Otsu, Japan) was used, as well as specific primers. U6 was used as a reference gene. After the initial denaturation at 95 °C for 15 min, the qPCR cycles were as follows: 40 cycles of denaturation at 94 °C for 15 s, annealing at 55 °C for 30 s and 70 °C for 30 s, reading of plate. Finally, the PCR was completed after 15 s at 60 °C with Roche LightCycler. The relative expression levels of miRNAs were normalized with U6 using the delta delta CT (2^−ΔΔCT^) method.

### 2.7. Statistical Analysis

Data were presented as means ± SEM. The Shapiro–Wilk test was first applied to confirm the normality of the data. When data followed a normal distribution, The Student’s *t*-test (unpaired) was used to compare two groups. Two-way analysis of variance (ANOVA) with Tukey’s post-hoc test was used for comparisons between multiple groups. One-way ANOVA was used for behavioral test of tail. Kruskal–Wallis, Student’s *t*-test and Mann–Whitney U tests were also carried out depending on whether the data showed normal distribution or not. *p*-values less than 0.05 were considered statistically significant. The GraphPad Prism program (version 8) was used to evaluate the data and plot the graphs.

## 3. Results

### 3.1. Behavioral Sex Differences Are Greater in Cc2d1a +/− Offspring of the Mouse Model for Autism

The groups of mice were produced in the *Balb/c* lineage at the same time and conditions (see Section 2). Two groups of offspring were produced by mating *Cc2d1a* +/− with the controls partner *Balb/c* (G1) or with a *Balb/c-Cc2d1a* +/− partner (G2) (heterozygous mutation of *Cc2d1a* gene) (see Section 2 and Supplementary Figure S1). The behaviors were clearly different in the *Cc2d1a* +/− offspring in the two groups obtained (G1 and G2) compared to the controls.

We compared parents and their offspring. Parents were tested after mating at four months of age, while offspring were tested at two months of age and virgin. Parents showed little to no difference from controls, but offspring showed a real difference (see figures).

**Figure 1:**
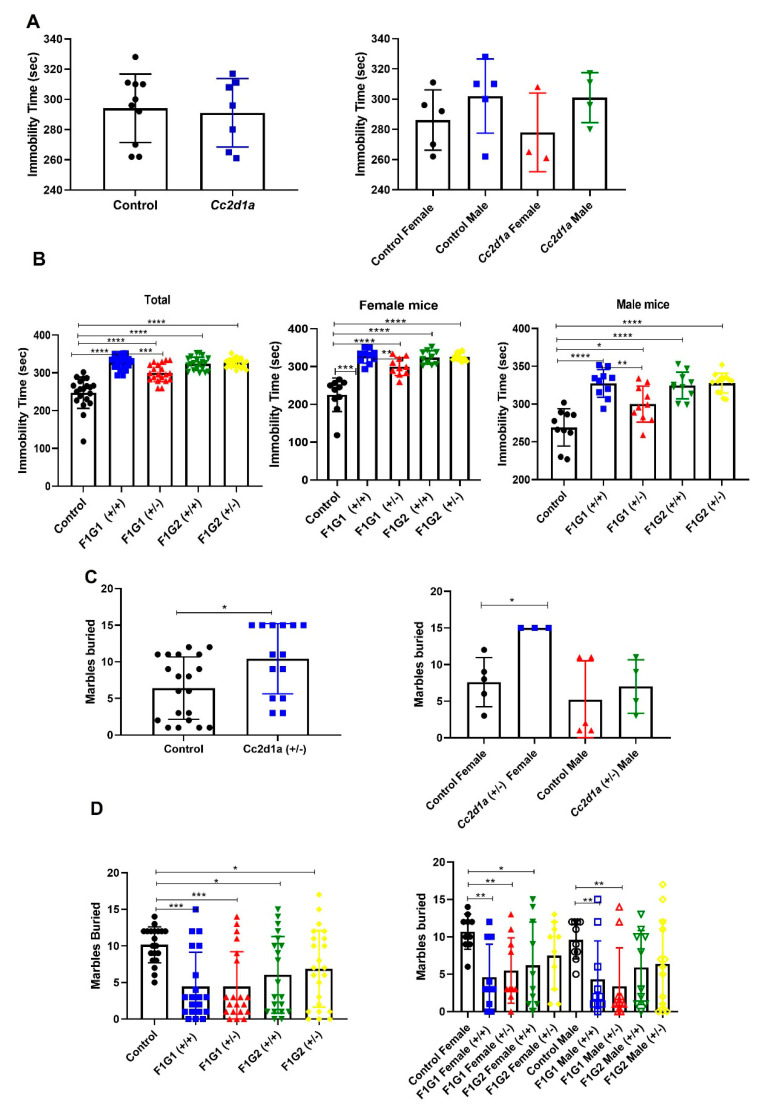

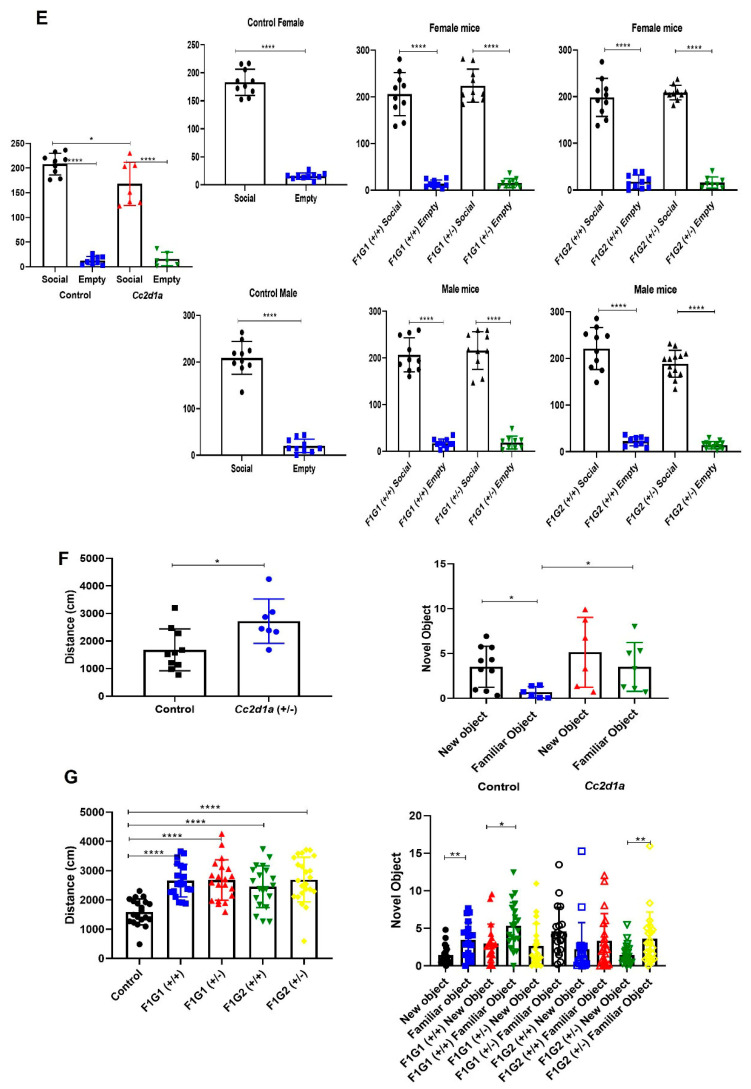

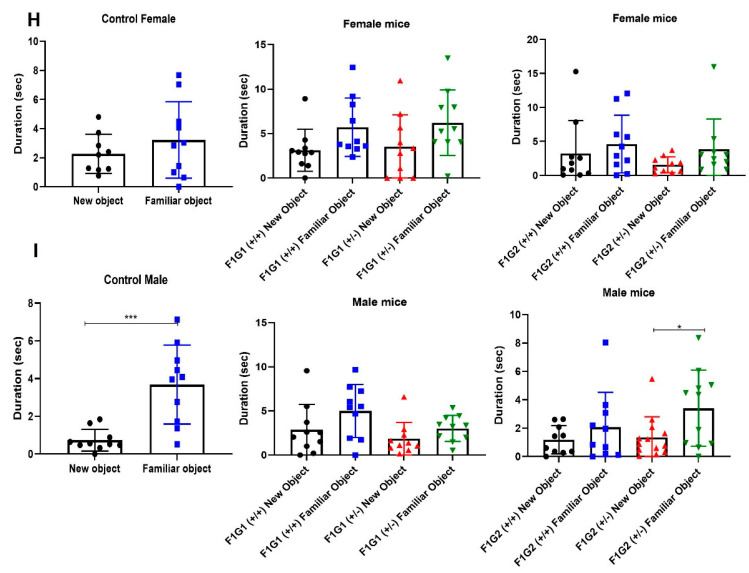
Tail suspension test for parent and F1 generation. (**A**). Test results for parents (n = 10, each sex). (**B**). Test results for F1 generation (n = 10, each sex). (**C**). Marble burying test for parents (n = 10, each sex). (**D**). Test results for F1 generation (n = 10, each sex). (**E**). Social interaction test for parents and F1 generation (n = 10, each sex). Total time was measured as sec. (**F**). Novel object recognition test for parents (n = 10, each sex). Distance was measured as cm. (**G**). Test results for F1 generation (n = 10, each sex). (**H**). Novel object recognition test for female mice (n = 10, each sex). Duration was measured as sec. (**I**). Novel object recognition test for male mice (n = 10, each sex). * *p* < 0.05, ** *p* < 0.01, *** *p* < 0.001, **** *p* < 0.0001.

Differences were found between in offspring groups by gender (Figure 1). Immobility times were longer in *Cc2d1a* +/− offsprings and therefore differed when the control group and the two *Cc2d1a* groups (G1 and G2) were compared (Figure 1). Immobility time also increased in progenies G1 and G2 +/+ and +/− genotypes compared to the progenies from control.

The number of buried beads differed significantly between the control group and the *Cc2d1a* progenies groups (*p* = 0.0158). The number of buried marbles were reduced in the progeny of the G1 and G2 +/+ and +/− genotypes groups compared to the control.

According to the social interaction test, there was no difference between the control and test groups. Both males and females spent more time in the empty cage, which was statistically significant (*p* < 0.0001; Figure 1E).

In the novel object test, the distance traveled differed significantly between the control and *Cc2d1a* groups (*p* = 0.0163, Figure 1F). The time spent with the novel object and the familiar object also differed. The distance traveled was higher in the G1 and G2 +/+ and +/− genotypes compared to the control and was statistically significant (*p* < 0.0001, Figure 1G). Differences were also seen between groups according to sex, with female offspring being more active in groups G1 and G2 (Figure 1H,I).

### 3.2. Sex-Dependent Distribution of Free or Hybrid/R-Loop miRNA Fractions in Cc2d1a +/− Offspring of the Mouse Model for Autism

**Figure 2:**
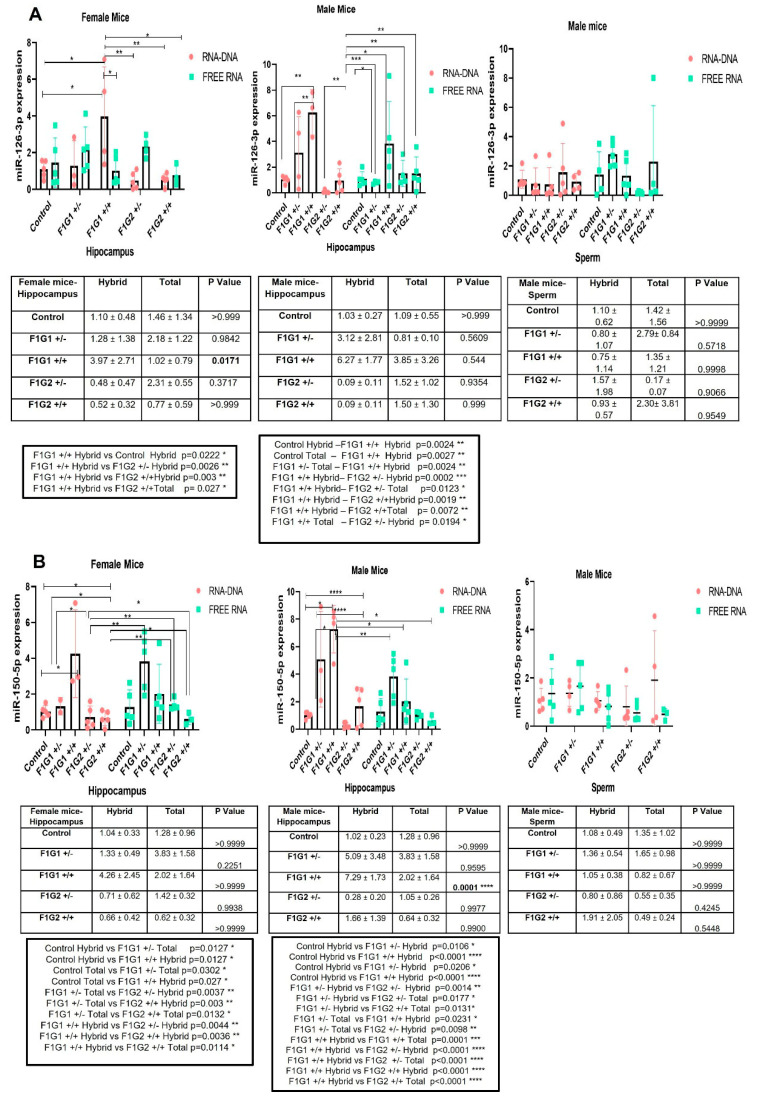

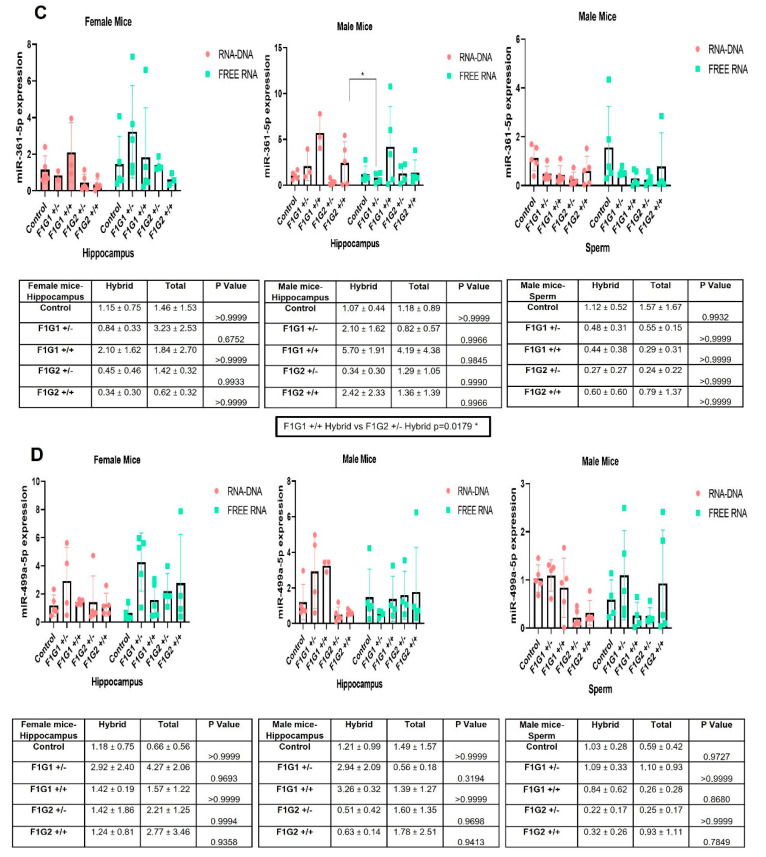

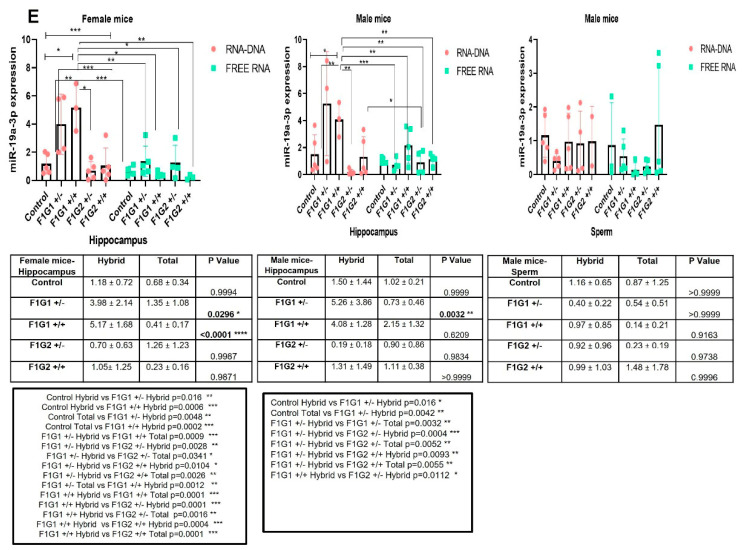
(**A**). miR-126-3p expression in hybrid and total groups. Graphs were shown for the female hippocampus, male hippocampus and male sperm. Data were expressed in mean ± standard deviation (n = 5, each group). (**B**). miR-150-5p expression in hybrid and total groups. Graphs were shown for the female hippocampus, male hippocampus and male sperm. Data were expressed in mean ± standard deviation (n = 5, each group). (**C**). miR-361-5p expression in hybrid and total groups. Graphs were shown for the female hippocampus, male hippocampus and male sperm. Data were expressed in mean ± standard deviation (n = 5, each group). (**D**). miR-499a-5p expression in hybrid and total groups. Graphs were shown for the female hippocampus, male hippocampus and male sperm. Data were expressed in mean ± standard deviation (n = 5, each group). (**E**). miR-19a-3p expression in hybrid and total groups. Graphs were shown for the female hippocampus, male hippocampus and male sperm. Data were expressed in mean ± standard deviation (n = 5, each group). * *p* < 0.05, ** *p* < 0.01, *** *p* < 0.001, **** *p* < 0.0001.

Figure 2 shows the results of miRNAs levels in the hippocampus (male and female) and in the spermatozoa cells in all groups (G1, G2 and controls; see above and Section 2). Results indicate tissue- and sex-dependent changes in the distribution of five miRNAs as fractions especially of RNA engaged in R-loops. The levels of differences are specific to a given miRNAs and tissues.

The expression profiles of miR-126-3p are shown in Figure 2A. Differences are observed in the hippocampus in both sexes. MiR-126-3p was higher in the free RNA fraction in the hippocampus of female mice, the expression of miR-126-3p is higher and significantly different in males in G1 and G2 in the hybrid fraction of RNA. In sperm, the free RNA fraction was higher in G1 and G2 (Figure 2A).

MiR-150-5p expression profiles are shown in Figure 2B. Higher expression of miR-150-5p is observed in female hippocampus in G1 and G2 in the free RNA fraction group, while a decrease is observed in all groups except G2 +/− in the male hippocampus (*p* < 0.0001). In sperm, there was a decrease in the free RNA fraction of the group compared to the hybrid, with the exception of F1G1 +/− (Figure 2B).

MiR-361-5p expression profiles are shown in Figure 2C. There was a decrease in F1G1 +/+ in the female hippocampus and an increase in all other groups overall. This difference was not significant compared to the hybrid (Figure 2C). In sperm, it increased in F1G1 +/− and F1G2 +/+ (Figure 2C).

MiR-499-5p expression profiles are shown in Figure 2D. There is an increase in miRNA levels in the female hippocampus in all groups, and this difference is not significant compared to the RNA fraction of in the hybrid. There was a decrease in G1 and an increase in G2 in the male hippocampus, different from that in females. In sperm, there was a decrease in F1G1 +/− and an increase in G2 which was not significant (Figure 2D).

The expression profiles of miR-19a-3p are shown in Figure 2E. In the female hippocampus, there was an increase in the level of miR-19a-3p transcripts in F1G1 +/− and a decrease in all other groups. The decreases observed in the F1G1 +/− and F1G1 +/+ groups were compared to the hybrid RNA fractions and the free fraction, and the result was significant (*p* = 0.03, *p* < 0.0001). There was an increase in F1G2 +/− animals in the male hippocampus and a decrease in the fraction of free RNA in all other groups. This difference was not significant compared to the hybrid, with the exception of F1G1 +/− (*p* = 0.0032). In sperm, it increased in F1G1 +/− and F1G2 +/+, and in other groups there was a decrease (Figure 2E).

## 4. Discussion

Neuronal development encompasses different stages from the embryo throughout neuronal life, primarily including the establishment and maintenance of transcription levels in mature adult brain cells [23]. However, the molecular mechanisms of adjustments in gene expression variations are still largely unknown, particularly subtle changes related to behavioral biology. In *Cc2d1a* heterozygous mice, we track Mendelian and non-Mendelian inheritance of autism, both of which induce variations in miRNAs levels. Here, we test the hypothesis of its molecular signature in the memory control of gene expression by testing the changes in hybrid formation between miRNA and DNA. These results are based on monitoring the behavior and expression level of five autism-related miRNAs in heterozygous and wild-type offspring. The hybrid RNA fractions attached to the DNA (R-loop) are compared. The quantity of these miRNAs varies by sex in the hippocampus compared to controls. In addition, their levels are reduced in sperm compared to the hippocampus and controls. These results constitute the first direct demonstration of (a) the differential localization of *miRNA* DNA/RNA hybrids in mouse brain cell models, (b) its involvement in mouse behavior and (c) sex differences along lineages.

Three key observations support these findings:

First, alterations in six miRNAs have previously been reported to be indicative of behavioral changes in autism in human and mouse models [7]. Additionally, microinjection of miRNAs into fertilized eggs alters their levels in the generated mice. Each of these miRNAs affects part of the complex phenotypes associated with autism syndrome in mice [24]. In our current experiments, we showed the differential amount of the same miRNAs in the free RNA fraction and/or the DNA/RNA hybrids fraction in heterozygous *Cc2d1a* model neuronal and germ cells.

We suggest that miRNA engaged in DNA/RNA hybrids may regulate their own transcriptional profiles in cells including neural cells. The formation of DNA/RNA hybrids with miRNA indicates the possibility of tissue-dependent self-controlled expression. Alteration of DNA secondary structures causes a higher proportion of damage during cell divisions, leading to an expansion of cellular damage [25]. It was also revealed that inappropriate DNA/RNA hybrids could affect gene expression [26]. Increased levels of miRNA engaged in DNA/RNA hybrids may have the ability to alter miRNA expression levels and, at the same time, subsequently significantly affect the levels of target transcripts.

Second, the results indicate that miRNA expression is affected in tissues compared to controls and is more pronounced in a sex-dependent manner in genetically modified *Cc2d1a* heterozygous mice. The prevalence of boys among ASD patients are striking. None of these miRNAs are located on the X chromosome. Other genetic protections could therefore be responsible for the rarity of autism in girls. However, our results here showed variation in behavior and miRNAs quantity in both sexes compared to controls, but still with differences between males and females in the mouse model. Our previous studies showed that the levels of five/(six) miRNAs, expressed in blood, hippocampus, testes and sperm differed in *Cc2d1a* heterozygous tissues [7]. In this regard, we reported that sustained alteration of six miRNAs in *Cc2d1a* heterozygous cells resulted in subsequent behavioral impairment and sex-specific partial autophagy [7,9].

Third, previous studies have shown that *miRNAs* are highly expressed in the CNS during embryonic development and in the peripheral nervous system [27]. Here, we show that miRNA expression is highly variable in regions where adult neurogenesis (hippocampus) continually occurs. We also demonstrated the presence of *miRNAs* in the purified DNA/RNA hybrid fractions. These results strongly suggest the possibility of a role for miRNA in the structure of DNA/RNA hybrids in the physiological functions of the CNS. Recently, the involvement of DNA/RNA hybrids in trinucleotide repeat neurologic diseases and the establishment of repressive histone modifications has been clearly reported [28,29,30]. However, direct evidence between R-loops and changes in miRNA expression require further study. Targeted deletions in vitro and in vivo are necessary to establish the details of the involvement of DNA/RNA hybrids.

These three key observations support the conclusion that miRNA is not only a post-transcriptional regulator, but also a key regulator of its own expression. Although we have not established the effects of miRNAs concentration on cellular memory, it seems interesting that a single short sequence can regulate both pre- and post-expression regulation [24]. A precedent for this phenomenon is observed in the case of TERRAs (Telomeric Repeat-containing RNA), present in the form of free RNA molecules in the nucleus but also in the form of DNA/RNA hybrids at the end of each chromosome. TERRA promotes telomere elongation, by associating with DNA, but also promotes telomerase activity [26,31].

In light of the many recent reports relating to the application of R-loops to disorders, it will be essential to understand in detail the roles of different miRNAs sequences in their own expression. In this regard, we propose that miRNA engaged in R-loop sensing provide a novel strategy to detect variations in gene expression for neurological changes. The expression levels of miRNAs are essential for tissue-specific identity. We know that miRNAs participate in the transcriptional regulation of several genes, including neuronal differentiation genes [32]. Impairment due to miRNAs tuning, triggered by binding of target RNAs to the enhancer region, is suggested to cause differentiation or division of precursor cells. For example, the concerted action of miRNAs and their localization would be important for inducing decisions regarding neural fate. miRNAs are involved in morphogenesis and patterning, and their proliferation-promoting roles are essential for cell maintenance and expansion of progenitor pools.

## 5. Limitations

Here, we tested parents (at four months and after mating) and offspring (virgin at two months). We observed differences between parents and their offspring in terms of behavior and miRNA expression. It is important to know the causes of these differences through future investigations. Although it is difficult to compare these results with autism in humans, we offer research tools that would be worth testing in families with autistic children.

We analyzed the variations of five of the six miRNAs detected in a previous study, gradually decreasing compared to patients’ relatives as a hereditary marker. Additionally, miRNAs levels can orchestrate variation in transcription of many target mRNAs and their translation during the developmental stage of tissues [33]. It will therefore be important to determine one by one the respective roles of these five miRNAs in controlling the outcome of mouse behaviors [24].

## 6. Conclusions

The unlimited variations in phenotypes within a given organism with the same genotype have always intrigued researchers. Deciphering the complexity of molecular mechanisms opens up perspectives for finding a coherent explanation. Here, for example, we report a short RNA as a transcriptional modulator of their specific locus. We propose a relatively simple and robust molecular tool to monitor these molecular variations according to phenotypes.

## Data Availability

The original contributions presented in the study are included in the article, further inquiries can be directed to the corresponding authors.

## Supplementary Materials

The following supporting information can be downloaded at: https://www.mdpi.com/article/10.3390/biom14091183/s1, Figure S1: Animal groups that conducted in this study and statistical analysis with F values.
