# Supplementary figures and images for: Autism-Related Cc2d1a Heterozygous Mice: Increased Levels of miRNAs Retained in DNA/RNA Hybrid Profiles (R-Loop)
Source: Biomolecules. 2024 Sep 20;14(9):1183. doi: 10.3390/biom14091183 (PMC11430583; doi:10.3390/biom14091183)

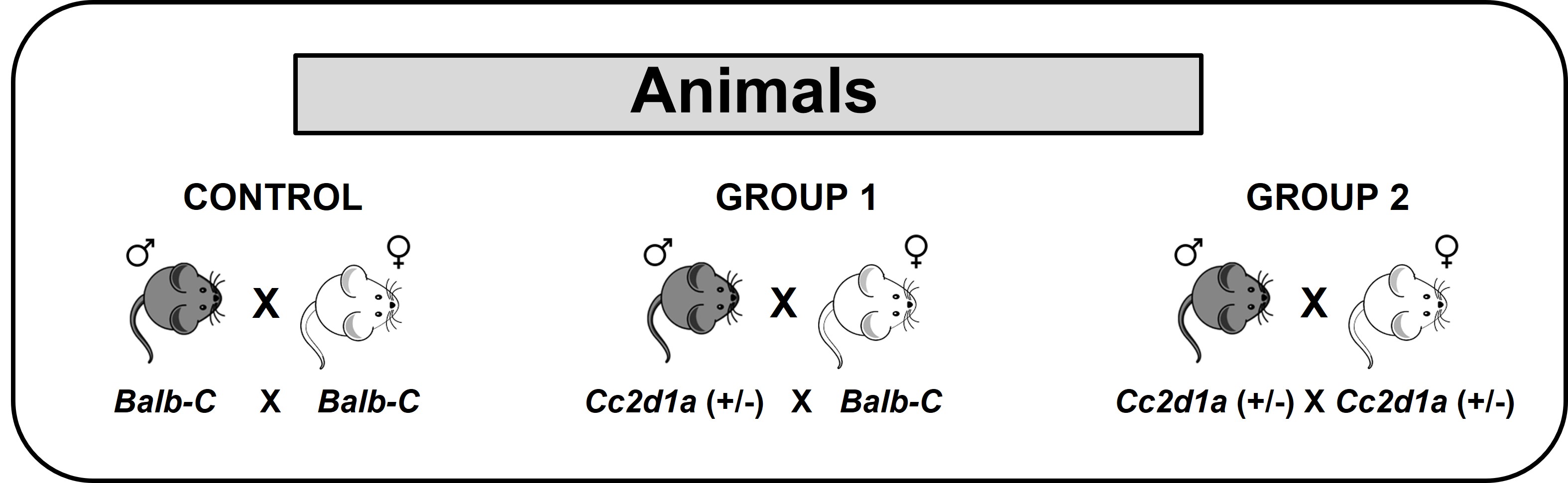

Supplement: Supplementary file 1 [file biomolecules-14-01183-s001.zip › supp figure.jpg]
